# Supplementary material for: Ballistic superconductivity and tunable π–junctions in InSb quantum wells
Source: Nat Commun. 2019 Aug 21;10:3764. doi: 10.1038/s41467-019-11742-4 (PMC6704170; doi:10.1038/s41467-019-11742-4)
Supplement: Supplementary file 1 — Supplementary Information [file 41467_2019_11742_MOESM1_ESM.pdf]

Supplementary Information for: Ballistic superconductivity and tunable π-junctions in InSb quantum wells

Chung Ting Ke,[1,*] Christian M. Moehle,[1,*] Folkert K. de Vries,[1]
Candice Thomas,[2,3] Sara Metti,[3,4] Charles R. Guinn,[2] Ray Kallaher,[3,5]
Mario Lodari,[1] Giordano Scappucci,[1] Tiantian Wang,[2,3] Rosa E. Diaz,[3]
Geoffrey C. Gardner,[3,5] Michael J. Manfra,[2,3,4,5,6] and Srijit Goswami[1,†]

[1]*QuTech and Kavli Institute of Nanoscience, Delft University of Technology, 2600 GA Delft, The Netherlands*

[2]*Department of Physics and Astronomy, Purdue University, West Lafayette, Indiana 47907, USA*

[3]*Birck Nanotechnology Center, Purdue University, West Lafayette, Indiana 47907, USA*

[4]*School of Electrical and Computer Engineering, Purdue University, West Lafayette, Indiana 47907, USA*

[5]*Microsoft Quantum at Station Q Purdue, Purdue University, West Lafayette, Indiana 47907, USA*

[6]*School of Materials Engineering, Purdue University, West Lafayette, Indiana 47907, USA*

SUPPLEMENTARY NOTE 1: WAFER GROWTH AND CHARACTERIZATION

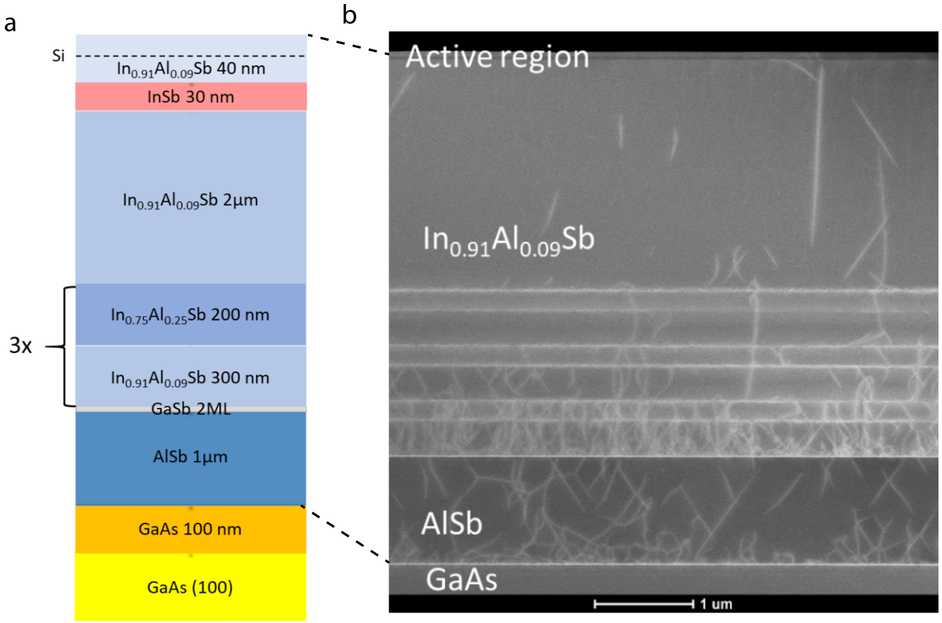

Supplementary Figure 1. **InSb quantum well.** (a), Layer stack of the InSb/GaAs heterostructure, where the layer constituents and thicknesses are indicated. (b), Scanning transmission electron micrograph of the structure of Supplementary Fig. 1a obtained in High Angle Annular Dark Field Mode along the [110] zone axis.

The wafer is characterized by measuring the (quantum) Hall effect in a Hall bar geometry at $T = 300$ mK. From a linear fit to the transversal resistance in a magnetic field range up to 1 T, we extract an electron density $n = 2.71 \cdot 10^{11}$ cm^{-2}, and by using the longitudinal resistivity at zero field, we obtain a mobility $\mu = 146,400$ cm^{2}V^{-1}s^{-1} (see Supplementary Table I). We calculate the corresponding mean free path to be $l_\mathrm{e} = 1.26$ μm. In Supplementary Table 1, we also include n, μ and l_e for the low mobility wafer, obtained from a quantum Hall measurement on this wafer. Data from the low mobility wafer is shown in Fig. 1d of the main text.

	High mobility wafer	Low mobility wafer
n (cm^{-2})	$2.71 \cdot 10^{11}$	$2.71 \cdot 10^{11}$
μ (cm^2/Vs)	146,400	61,500
l_e (μm)	1.26	0.53

Supplementary Table I. Electron density, mobility and mean free path for the high and low mobility wafer, obtained from quantum Hall measurements at $T = 300$ mK.

SUPPLEMENTARY NOTE 2: MULTIPLE ANDREEV REFLECTIONS AND EXCESS CURRENT

To further characterize the superconductivity in our JJs, we study multiple Andreev reflections (MAR) in a representative JJ, by measuring its differential resistance, $\mathrm{d}V/\mathrm{d}I$, as a function of applied bias voltage, V. In Supplementary Fig. 2a, we observe three dips in $\mathrm{d}V/\mathrm{d}I$, the first, at 2Δ, corresponding to the coherence peaks of the superconducting density of states, and two MAR peaks at $2\Delta/2$ and $2\Delta/3$. From these peaks we extract an induced superconducting gap $\Delta = 0.9$ meV. In addition, we estimate the transparancy

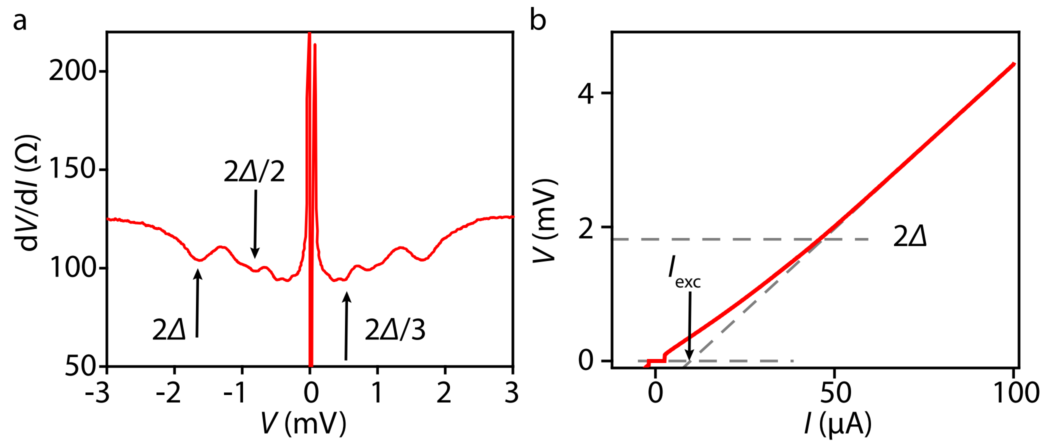

Supplementary Figure 2. **Josephson junction characterization.** (a), Differential resistance, $\mathrm{d}V/\mathrm{d}I$, as a function of bias voltage, V, showing multiple Andreev reflections. Three dips at $V = 2\Delta$, $2\Delta/2$ and $2\Delta/3$ are highlighted. (b), Voltage measured as a function of bias current. The excess current, I_{exc}, and $V = 2\Delta$ are indicated.

of the same JJ by measuring its excess current, I_{exc}, and normal state resistance, R_{n}. This measurement is shown in Supplementary Fig. 2b, where we perform a linear fit in the high bias region of the $I-V$ curve ($V > 2\Delta$) and obtain $I_{\text{exc}} = 9\ \mu$A and $R_{\text{n}} = 50\ \Omega$. Using the OBTK model [1], we find a value of 0.62 for the transparency of the JJ.

These transparencies are moderate compared to, for example, hybrid devices made with epitaxial interfaces between Aluminum and InAs 2DEGs [2]. In fact, recent work has shown that high quality interfaces can even be made between Aluminum and InSb wires [3–5]. We expect that similar materials developments with InSb 2DEGs would enable strong proximity coupling, an important requirement for exploring topological superconductivity in these systems.

SUPPLEMENTARY NOTE 3: WEAK ANTI-LOCALIZATION AND SPIN-ORBIT INTERACTION ENERGY

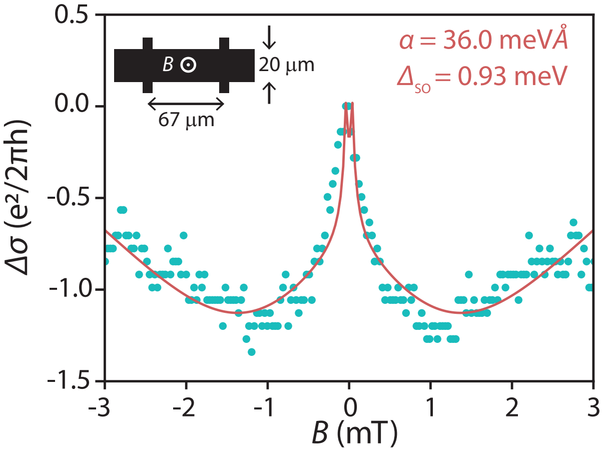

Supplementary Figure 3. **Weak anti-localization analysis.** Measured longitudinal conductivity difference, $\Delta\sigma$, as a function of magnetic field, B, displaying a weak anti-localization peak around zero field. We fit (red) the data (cyan) using the ILP model and extract the SOI energy at the Fermi energy, Δ_{SO}, from which we calculate the Rashba spin-orbit parameter α. The inset shows a schematic of the Hall bar device, indicating its length and width, and the magnetic field direction.

To obtain an estimate of the typical energy scale associated with the spin-orbit interaction, we performed weak anti-localization (WAL) measurements. We use a Hall bar device

(inset Supplementary Fig. 3) fabricated on the high mobility wafer (Supplementary Fig. 1), and apply magnetic field perpendicular to the Hall bar. The measurement in Supplementary Fig. 3 reveals the typical WAL peak around zero field. This peak is caused by suppression of coherent backscattering at small magnetic fields due to the spin-orbit interaction. As we expect the Dyakonov Perel scattering mechanism to be dominating in our high mobility wafer, we use the theory developed by Iordanskii, Lyanda-Geller and Pikus [6] to fit the data:

$$\frac{\Delta\sigma(B)}{e^2/2\pi h} = -\frac{1}{a} - \frac{2a_0 + 1 + H_{\rm s}}{a_1(a_0 + H_{\rm s} - 2H_{\rm s})} - 2\ln H_{\rm tr} - \Psi\left(1/2 + H_\phi\right) - 3C$$
$$+ \sum_{n=1}^{\infty}\left[\frac{3}{n} - \frac{3a_{\rm n}^2 + 2a_{\rm n}H_{\rm s} - 1 - 2(2n+1)H_{\rm s}}{(a_{\rm n} + H_{\rm s})a_{\rm n\text{-}1}a_{\rm n+1} - 2H_{\rm s}[(2n+1)a_{\rm n} - 1]}\right],$$

where Ψ is the Digamma function, C the Euler constant, and

$$a_{\rm n} = n + \frac{1}{2} + H_\phi + H_{\rm s} \qquad H_{{\rm tr},\phi,{\rm s}} = \frac{\hbar}{4eDB\tau_{{\rm tr},\phi,{\rm s}}} \qquad \Delta_{\rm SO} = \sqrt{\frac{2\hbar^2}{\tau_{\rm tr}\tau_{\rm s}}},$$

with $D = v_{\rm F}l_e/2$, and $\tau_{{\rm tr},\phi,{\rm s}}$ the scattering times for elastic, inelastic and spin-orbit scattering, respectively. We find a spin-orbit energy splitting at the Fermi level ($\Delta_{\rm SO}$) of 0.93 meV. The Rashba spin-orbit parameter of $\alpha = 36$ meVÅ is calculated following $\alpha = \Delta_{\rm SO}/k_{\rm F}$, where k_F is deduced from a classical Hall measurement. Finally, we compare $\Delta_{\rm SO}$ to the Zeeman energy. For a Landé g-factor of 25, $\Delta_{\rm SO} > E_{\rm Z}$ up to 640 mT. We are therefore in the spin-orbit dominated regime for the $0-\pi$ transition.

SUPPLEMENTARY NOTE 4: MAGNETIC FIELD ALIGNMENT

To ensure we are sweeping the magnetic field in the plane of the JJs only, we characterize the misalignment of our vector magnet axes, $B'_{\rm y}$ and $B'_{\rm z}$, used to apply the magnetic field in-plane and out-of-plane of the JJ, $B_{\rm y}$ and $B_{\rm z}$. In Supplementary Fig. 4a we present a systematic measurement of the Fraunhofer interference pattern induced by $B'_{\rm z}$ with increasing $B'_{\rm y}$. We track the magnetic field at which the central lobe reaches its maximum $I_{\rm s}$, $B'_{\rm z,max}$ and plot this for all $B'_{\rm y}$ in Supplementary Fig. 4b. The linear dependence observed, represents a small misalignment angle of $\theta = 1.4°$. We take this angle into account when sweeping the in-plane field, $B_{\rm y} = \cos(\theta)B'_{\rm y} + \sin(\theta)B'_{\rm z}$, and disregard it for the out-of-plane direction, $B_{\rm z} = B'_{\rm z}$.

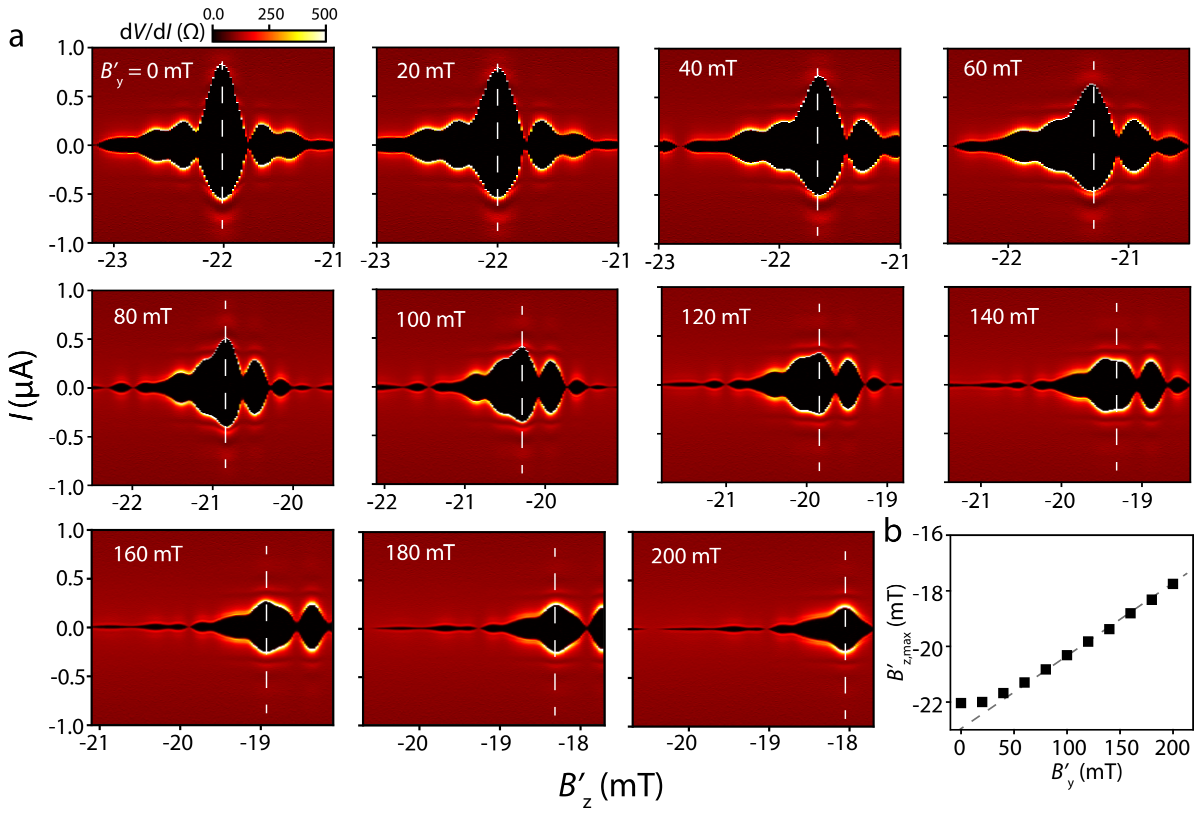

Supplementary Figure 4. **Field alignment.** (a), Differential resistance, $\mathrm{d}V/\mathrm{d}I$ maps as a function of current bias, I, and out-of-plane magnetic field, B'_{z}, with increasing in-plane magnetic field, B'_{y}, in steps of 20 mT. We track the central lobe of the interference pattern, labeled by white dash lines, to obtain $B_{\mathrm{z,max}}$. (b), The $B'_{\mathrm{z,max}}$ vs. B'_{y} dependence showing the small perpendicular component of B'_{y}.

At larger magnetic fields the patterns become asymmetric. This asymmetry has two possible origins which we expect to coexist in our samples. The first is the effect of magnetic vortices which nucleate in our type II superconductor (NbTiN) at moderate magnetic fields. The second has to do with terms in the Hamiltonian that break mirror symmetry of the potential in the JJ (e.g., small amounts of disorder at the interface) [7]. To ensure that these asymmetries do not influence our extraction of I_{s} or the magnetic field at which it revives, we performed two separate cooldowns (Fig. 2a in the main text) and confirmed that the results are in agreement. The large offset observed at zero in-plane magnetic field is a trivial offset from the magnet power supply which we have corrected for in Fig. 1c of the main text.

SUPPLEMENTARY NOTE 5: IN-PLANE INTERFERENCE CONSIDERATIONS

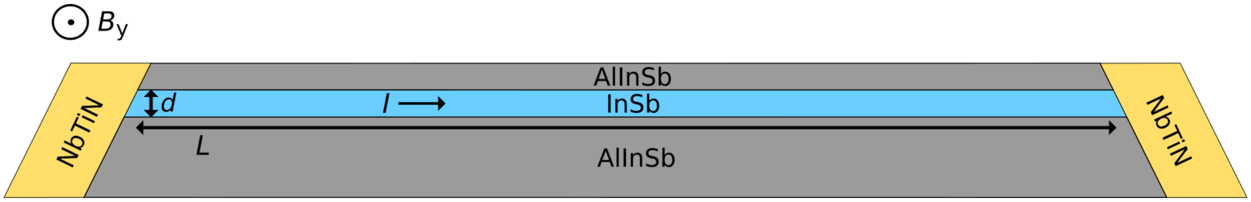

Supplementary Figure 5. **Schematic of quantum well.** Cross-sectional illustration of the InSb quantum well for a JJ with $L = 1.1$ μm and $d = 30$ nm. The image is drawn to scale and the in-plane magnetic field direction, B_y is indicated.

We observe a switching current, I_s modulation in a JJ with $L = 1.1$ μm, with minima at 470 mT and 1250 mT, which are attributed to Zeeman induced $0-\pi$ transitions. One might be inclined to believe that this modulation is caused by an in-plane Fraunhofer interference effect, due to the finite thickness ($d = 30$ nm) of the InSb quantum well. The I_s minima of such a Fraunhofer pattern are expected to occur at $B_\mathrm{node} = \mathrm{N}\Phi_0/A$, where Φ_0 is the magnetic flux quantum, $A = d \cdot L$ is the cross-sectional 2DEG area and $\mathrm{N} = 1, 2, 3, ...$ The second minimum should thus occur at twice the value of the first, which is not the case here. Moreover, based on the estimated cross-sectional area of the JJ (see Supplementary Fig. 5), one would expect the first node to be at 60 mT, inconsistent with the observation.

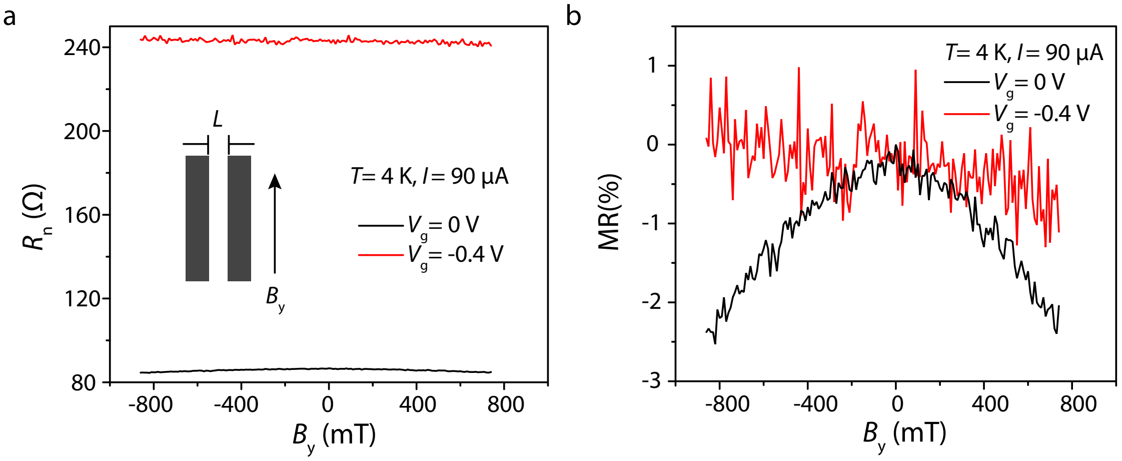

Supplementary Figure 6. **In-plane magnetoresistance.** (a), Normal state resistance (R_n) of JJ ($L = 1.1$ μm) as a function of B_y at two different gate voltages. (b), Calculated MR (in %).

In fact, it has been shown [8, 9] that a oscillatory interference pattern is not expected at all in such an SNS junction with $L \gg d$. Finally, for an in-plane interference effect one expects the B value at which the I_s minima occur to increase for more negative gate voltages, since the wavefunction is then squeezed and d effectively reduced. However, we observe the opposite behavior (i.e., the minima move to lower B), as expected for Zeeman-induced $0{-}\pi$ transitions. To conclude, we rule out an in-plane interference effect as a possible explanation for the supercurrent modulation.

We also performed measurements of the normal state resistance (R_n) as a function of in-plane magnetic field B_y to rule out possible magnetoresistance (MR) effects as a cause for the observed modulation in supercurrent (see Supplementary Fig. 6). To eliminate any remnant effects of superconductivity, the measurements are performed at high temperature (4 K) and high DC current bias (90 μA). We see a small MR (a few %) only at the highest density ($V_\mathrm{g} = 0$ V), however there is no correlation with the supercurrent modulation (see Fig. 2 in main text), which changes by almost two orders of magnitude in the same magnetic field range.

SUPPLEMENTARY NOTE 6: ADDITIONAL GATE-DRIVEN $0{-}\pi$ TRANSITIONS AND ERROR ANALYSIS

Here, we present additional data of the gate-driven $0 - \pi$ transitions in the JJ with $L = 1.1$ μm. The gate voltages of the $0 - \pi$ transitions presented in the phase diagram are extracted from the plots in Supplementary Fig. 7. To systematically extract the value where gate-driven $0 - \pi$ transition occurs and its error, we use a fit of the linetraces from Supplementary Fig. 7, at zero I. At the transition point, a peak in $\mathrm{d}V/\mathrm{d}I$ indicates the $0{-}\pi$ transition. As an example, we show a single linetrace at 310 mT in Supplementary Fig. 8a, and extract the standard deviation, σ, based on a Gaussian fit of the peak. Subsequently, we used the gate to density mapping to convert σ to the error bar shown in the phase diagram. This fitting procedure is used for all magnetic fields (Supplementary Fig. 8b).

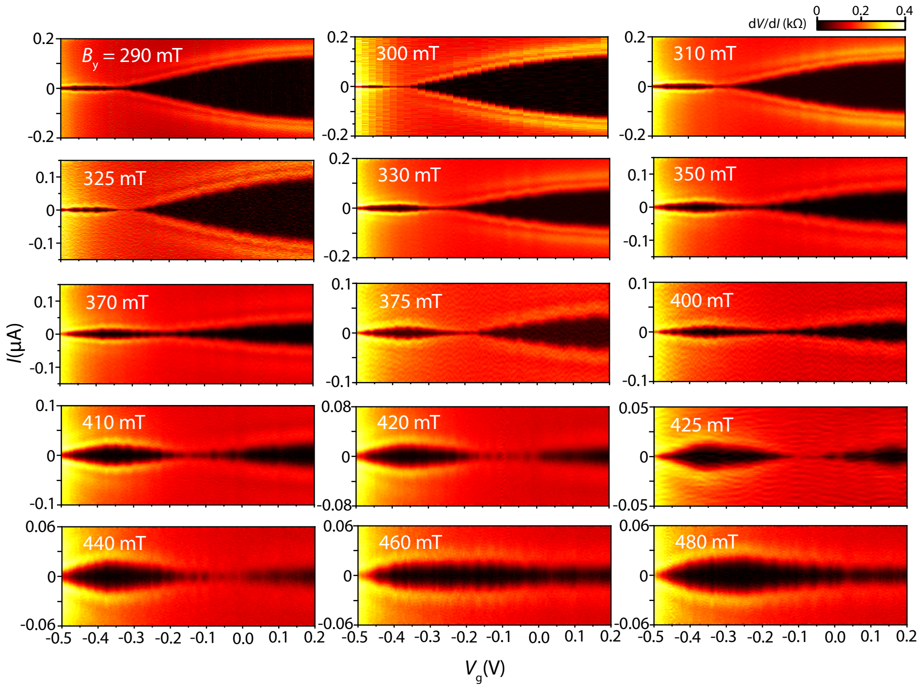

Supplementary Figure 7. **Additional gate-driven $0-\pi$ transitions.** Differential resistance, $\mathrm{d}V/\mathrm{d}I$, as a function of current bias, I, and gate voltage, V_g, for the in-plane magnetic field values, B_y, indicated.

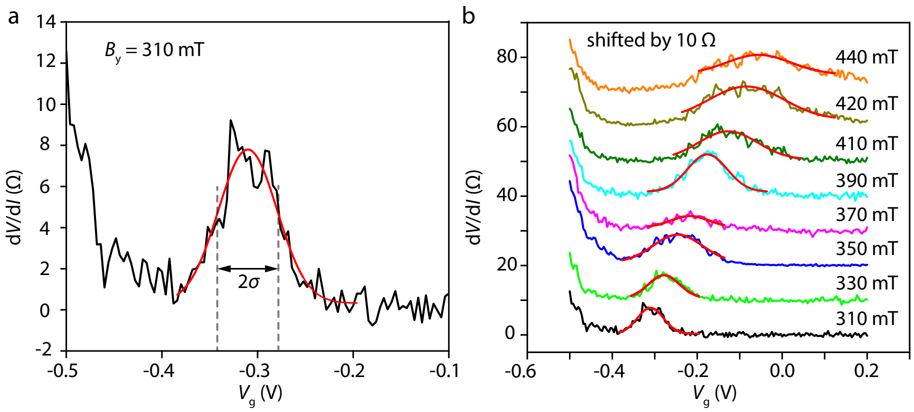

Supplementary Figure 8. **Transition point extraction and error analysis.** (a-b), Linetraces of the differential resistance, $\mathrm{d}V/\mathrm{d}I$, in the JJ with $L = 1.1\ \mu$m as a function of gate voltage, V_g, for magnetic fields, B_y of 310 mT for (a), and as indicated for (b), respectively. The peaks observed are fitted with a Gaussian curve, to obtain the standard deviation, σ. In (b) the traces are shifted for clarity.

SUPPLEMENTARY NOTE 7: EFFECTIVE MASS MEASUREMENT

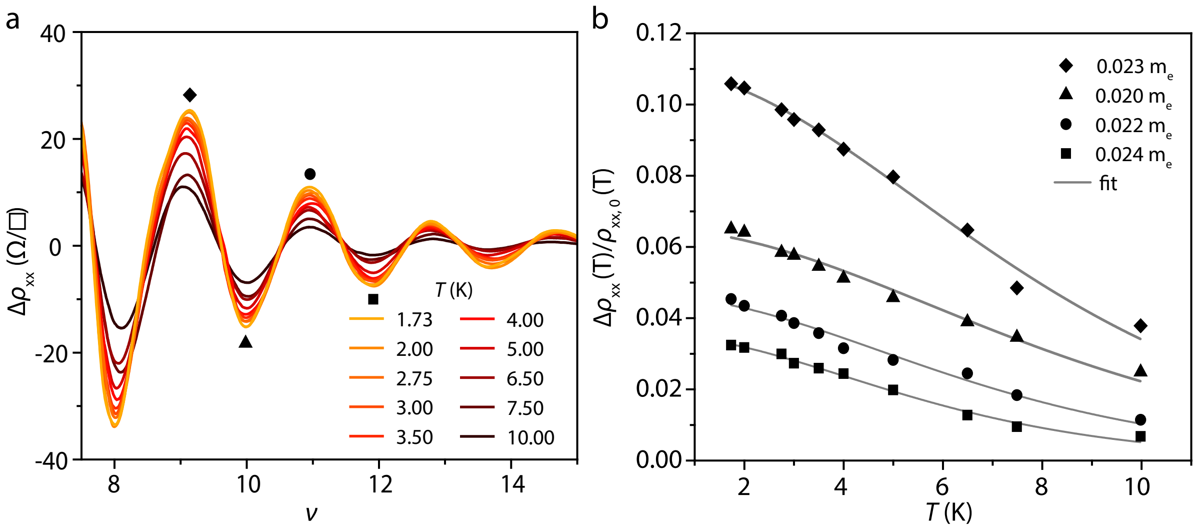

Supplementary Figure 9. **Temperature dependence of Shubnikov-de Haas oscillations and fitting for effective mass.** (a), Shubnikov-de Haas oscillation amplitude after polynomial background subtraction as a function of filling factor for temperatures $T = 1.73 - 10$ K. The symbols denote points that are used to extract the effective mass. (b), Temperature dependence of the oscillation amplitude (symbols). The solid lines are fits to the data (using Eq. 1) in order to obtain the effective mass.

To extract the effective mass of the electrons in the InSb 2DEG, the temperature dependence of the Shubnikov-de Haas (SdH) oscillation amplitude is measured in a Hall bar geometry. Supplementary Figure 9a shows the magnetoresistance oscillations after the subtraction of a polynomial background, $\Delta\rho_{\rm xx}$, as a function of filling factor, ν, for temperatures ranging from $T = 1.73$ K to $T = 10$ K. At a fixed filling factor, the effective mass, m^*, can be obtained from a fit to the damping of the SdH oscillation amplitude with increasing temperature, using the expression

$$\frac{\Delta\rho_{\rm xx}(T)}{\rho_{\rm xx,0}(T)} \propto \frac{\alpha T}{\sinh(\alpha T)} \ , \tag{1}$$

where $\rho_{\rm xx,0}(T)$ is the temperature-dependent low-field resistivity and $\alpha = \pi k_{\rm B} m^* \nu/(\hbar^2 n)$. Supplementary Figure 9b shows such fits to the oscillation minima and maxima of $\nu = 10$ and $\nu = 12$, resulting in a mean effective mass of $m^* = (0.022 \pm 0.002) \cdot m_e$, with m_e being the free electron mass.

---

* Equal contributions

† S.Goswami@tudelft.nl

[1] K. Flensberg, J. B. Hansen, and M. Octavio, Phys. Rev. B **38**, 8707 (1988).

[2] M. Kjaergaard, H. J. Suominen, M. P. Nowak, A. R. Akhmerov, J. Shabani, C. J. Palmstrøm, F. Nichele, and C. M. Marcus, Phys. Rev. Applied **7**, 034029 (2017).

[3] H. Zhang, Ö. Gül, S. Conesa-Boj, M. P. Nowak, M. Wimmer, K. Zuo, V. Mourik, F. K. de Vries, J. van Veen, M. W. A. de Moor, J. D. S. Bommer, D. J. van Woerkom, D. Car, S. R. Plissard, E. P. A. M. Bakkers, M. Quintero-Pérez, M. C. Cassidy, S. Koelling, S. Goswami, K. Watanabe, T. Taniguchi, and L. P. Kouwenhoven, Nat. Commun. **8**, 16025 (2017).

[4] H. Zhang, C.-X. Liu, S. Gazibegovic, D. Xu, J. A. Logan, G. Wang, N. van Loo, J. D. S. Bommer, M. W. A. de Moor, D. Car, R. L. M. Op het Veld, P. J. van Veldhoven, S. Koelling, M. A. Verheijen, M. Pendharkar, D. J. Pennachio, B. Shojaei, J. S. Lee, C. J. Palmstrøm, E. P. A. M. Bakkers, S. D. Sarma, and L. P. Kouwenhoven, Nature **556**, 74 (2018).

[5] S. T. Gill, J. Damasco, B. E. Janicek, M. S. Durkin, V. Humbert, S. Gazibegovic, D. Car, E. P. A. M. Bakkers, P. Y. Huang, and N. Mason, Nano Lett. **18**, 6121 (2018).

[6] S. V. Iordanskii, Y. B. Lyanda-Geller, and G. E. Pikus, Zh. Eksp. Teor. Fiz. **60**, 199 (1994).

[7] A. Rasmussen, J. Danon, H. Suominen, F. Nichele, M. Kjaergaard, and K. Flensberg, Phys. Rev. B **93**, 155406 (2016).

[8] R. Monaco, M. Aaroe, J. Mygind, and V. P. Koshelets, Phys. Rev. B **79**, 144521 (2009).

[9] F. Chiodi, M. Ferrier, S. Guéron, J. C. Cuevas, G. Montambaux, F. Fortuna, A. Kasumov, and H. Bouchiat, Phys. Rev. B **86**, 064510 (2012).
